# Modified Tumor Budding as a Better Predictor of Lymph Node Metastasis in Early Gastric Cancer: Possible Real-World Applications

**DOI:** 10.3390/cancers13143405

**Published:** 2021-07-07

**Authors:** Kwangil Yim, Won Mo Jang, Sung Hak Lee

**Affiliations:** 1 Department of Hospital Pathology, College of Medicine, The Catholic University of Korea, Seoul 06591, Korea; kangse_manse@catholic.ac.kr; 2 Department of Public Health and Community Medicine, Seoul Metropolitan Government-Seoul National University Boramae Medical Center, Seoul 07061, Korea; thomasj@snu.ac.kr

**Keywords:** tumor budding, modified tumor budding, early gastric cancer, lymph node metastasis, endoscopic resection, surgical indication, assessment method

## Abstract

**Simple Summary:**

To obtain the optimal treatment effect of endoscopic resection (ER) in early gastric carcinoma (EGC), a well-established indication for post-ER surgery is needed. In addition, accurate prediction of lymph node metastasis (LNM) is necessary to achieve this goal. Here, we present modified tumor budding (mTB), which excludes signet ring cells from conventional tumor budding (TB) as a novel predictor for LNM. Conventional TB and mTB were the most predictive independent factors for LNM. Furthermore, mTB was superior to TB in predicting LNM (*p* = 0.0004–0.0008). In conclusion, mTB significantly enhanced the predictive power of LNM, which could be a novel indicator for determining post-ER surgery.

**Abstract:**

Endoscopic resection (ER) is a minimally invasive treatment for early gastric cancer (EGC) with a low risk of lymph node metastasis (LNM). Recently, tumor budding (TB) has emerged as a potential predictor of LNM in EGC. We assessed the clinical significance of modified TB (mTB) that excludes the signet ring cell component and compared several TB assessment methods. Two hundred and eighty-nine patients with EGC at Uijeongbu St. Mary’s Hospital from 2010 to 2021 were enrolled. In univariate analysis, age, size, depth of invasion, tumor type, histologic type, Lauren classification, lymphatic invasion, venous invasion, poorly differentiated carcinoma (“not otherwise specified” predominant), and TB were significantly associated with LNM. Multivariate regression analysis showed that mTB (difference area under the curve [dAUC] = 0.085 and 0.087) was superior to TB (dAUC = 0.054 and 0.057) in predicting LNM. In addition, total TB counts on representative slide sections (dAUC = 0.087 and 0.057) in assessing TB and mTB and the ITBCC method (dAUC = 0.085) in mTB were superior to the presence or absence method (dAUC = 0.042 and 0.029). The mTB significantly increases LNM prediction ability, which can provide important information for patients with EGC.

## 1. Introduction

Endoscopic resection (ER) has been used as a minimally invasive treatment method for early gastric cancer (EGC) [1,2] because of its lower incidence of complications [3,4] and shorter hospitalization periods than surgery [5]. According to the Japanese Gastric Cancer Association [1], GC lesions that are technically feasible for ER can be divided into three categories based on the risk of lymph node metastasis (LNM): absolute indication, expanded indication, and relative indication. Absolute indication is defined as a tumor that can undergo ER as a standard treatment with an LNM risk of less than 1%. The expanded indication was designed to apply ER to an undifferentiated-type adenocarcinoma; the LNM risk for the expanded indication may be less than 1%, but sufficient evidence is lacking [1]. However, despite these criteria for ER [1], extragastric recurrence after ER was reported in 0.14–0.21% of cases [6,7,8]. Conversely, the possibility of ER implementation remains open to more patients with relative indications [1,9]. In relative indications (i.e., a category other than absolute or expanded indication) tumors usually need to be treated with surgery, but ER may still lead to a cure. Therefore, ER could be an option considering various clinical circumstances [1].

Tumor budding (TB) is a well-known risk factor for LNM in early-stage colorectal cancer [10,11,12,13]. Recently, it has also emerged as a potential predictor of LNM in gastric cancer (GC) [12,14,15,16,17,18,19]. However, studies on the association between TB and LNM in GC have been conducted mainly in intestinal-type carcinoma [12,14,19], as it is difficult to discern TB in poorly cohesive carcinoma (PCC) cases because of its discohesive pattern [16,20]. Other studies have demonstrated that TB is a risk factor for LNM in all pT stages [14,15,16], in submucosal EGCs [17] or when dividing tumors into those with and without TB [17,18]. Therefore, further studies focusing on TB evaluation methods available for both intestinal/diffuse types and intramucosal/submucosal GCs are needed. Further research on the standardized assessment of TB and in a group that incorporates all kinds of EGCs, such as intramucosal/submucosal and intestinal/diffuse-type EGCs, is needed.

In the present study, we compared conventional TB and modified TB (mTB), which excludes signet ring cells (SRCs), to overcome the difficulties of TB assessment in PCC [19] on the basis that SRC carcinoma (SRCC) has shown favorable survival in EGC [21,22], and poorly cohesive carcinoma not otherwise specified (PCC-NOS) is more strongly associated with poor prognosis and LNM than SRCC [23]. In addition, TB was evaluated using the criteria proposed by the International Tumor Budding Consensus Conference (TB-ITBCC) [24] and the presence/absence of TB (TB-YN) method as well as the total number of tumor buds on a whole slide (total-TB). This is because the traditional method of evaluating TB (i.e., peritumoral budding (PTB)) is difficult to apply in mucosal GC due to the lack of clear demarcation of the invasive front, and TB is reported to be correlated with LNM in both intratumoral budding (ITB) and PTB [12,24,25].

## 2. Materials and Methods

### 2.1. Patient and Clinicopathological Data

A total of 289 patients with EGC who had undergone radical gastrectomy regardless of previous ER in Uijeongbu St. Mary’s Hospital between January 2010 and January 2021 were enrolled in this study. Clinicopathological parameters including the total number of lymph nodes, age, sex, tumor location, gross type, size, depth of invasion, differentiation, Lauren classification, and LNM were reviewed retrospectively from medical records. The histological type was collected and divided into six main subtypes: tubular, papillary, mucinous, SRCC, PCC-NOS, and mixed adenocarcinoma according to the WHO classification [26]. No other rare subtypes were found in the enrolled patients. This study was approved by the Institutional Review Board of the College of Medicine at the Catholic University of Korea (XC20RIDI0155).

### 2.2. Histopathologic Analysis

Hematoxylin and eosin stained tumor sections were evaluated for the presence or absence of ulcers, lymphatic invasion, venous invasion, poorly differentiated carcinoma not otherwise specified predominant cluster (PNC) and TB/mTB (by TB-ITBCC, TB-YN and total-TB).

### 2.3. Lymphatic and Venous Invasion

The diagnosis of lymphatic and venous invasion was summarized in a previous study [11]. Briefly, lymphatic invasion is defined as the presence of tumor cell clusters within the lymphatic space lined by a single layer of endothelial cells with no evidence of blood vessels. Similarly, we defined vascular invasion as tumor cell nests in spaces lined by endothelium and filled with red blood cells.

### 2.4. Ulcers

In this study, an ulcer was defined as a break in the mucous epithelium, especially the muscularis mucosae.

### 2.5. TB and mTB: SRCC and PCC-NOS

SRCC is defined as a tumor that is predominantly composed of SRCs with optically clear cytoplasmic mucin and an eccentrically placed nucleus. PCC-NOS is composed of all other poorly cohesive not otherwise specified (PC-NOS) cancer cells that are mismatched to the morphology of SRC [26,27].

TB has traditionally been defined as isolated single cancer cells or <5 cancer cells in the invasive front [12,24]. Some authors renamed the traditional TB concept as PTB and newly defined ITB as when TB is in the center of the tumor [28].

**Figure 1:**
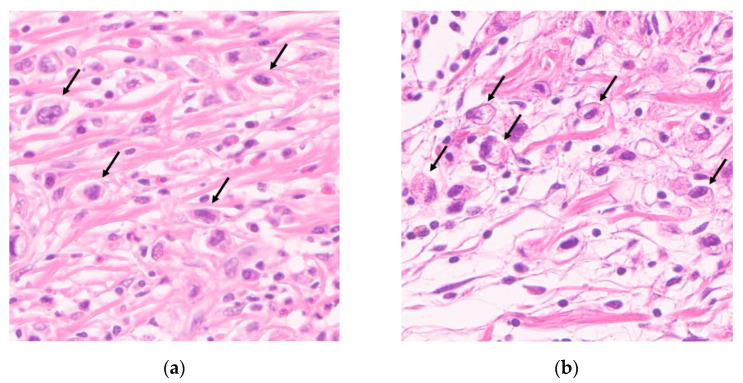
Representative histopathological images of tumor budding (magnification ×400). (**a**) Hematoxylin and eosin staining of a tumor section showing tumor budding morphologically consistent with poorly cohesive carcinoma not otherwise specified and (**b**) signet ring cell carcinoma.

We defined mTB as a novel pathologic factor that excludes SRC from conventional TB. When counting conventional TB, we measured all TB cells consistent with SRC and PC-NOS cells, and we measured only TB cells morphologically consistent with PC-NOS cells (Figure 1a,b). This is because diffuse-type EGC showed borderline poor survival, whereas intestinal-type EGC showed definite poor survival in a high TB group compared to a low TB group [19]. Moreover, SRCC had a better prognosis than intestinal-type EGC [21,22], and PCC-NOS showed poor prognosis and more LNM than SRCC [23].

### 2.6. TB Assessments: Presence/Absence of TB (TB-YN), Method Proposed by ITBCC (TB-ITBCC), Total Number of Tumor Buds on a Whole Slide (Total-TB)

First, TB-YN was used to divide tumors into two groups depending on the presence or absence of TB. Second, TB was assessed using the method described by ITBCC (TB-ITBCC). After selecting a hotspot by scanning 10 fields at ×100 magnification, we counted the tumor buds in the selected hotspot. Then, to adjust the TB count to 0.785 mm^2^, the count was converted by applying the normalization factor corresponding to the eyepiece field number [24]. The final method, total-TB, assessed the total number of tumor buds in a whole slide regardless of whether TB was on the invasive front or not. By using total-TB, both traditional PTB and ITB could be counted.

### 2.7. Poorly Differentiated Cluster (PDC) and PCC-NOS Predominant Cluster (PNC)

In colorectal carcinoma, PDCs, defined as cancer cell clusters of ≥5 carcinoma cells without gland formation, has been reported as a high-risk factor for poor prognosis and LNM [11,29,30]. PDCs can be easily recognized in intestinal-type GC, but not in PCC. Moreover, PDC, by definition, cannot discriminate between SRCC and PCC-NOS, although the mutational patterns and clinical outcomes are different in both [21,23].

**Figure 2:**
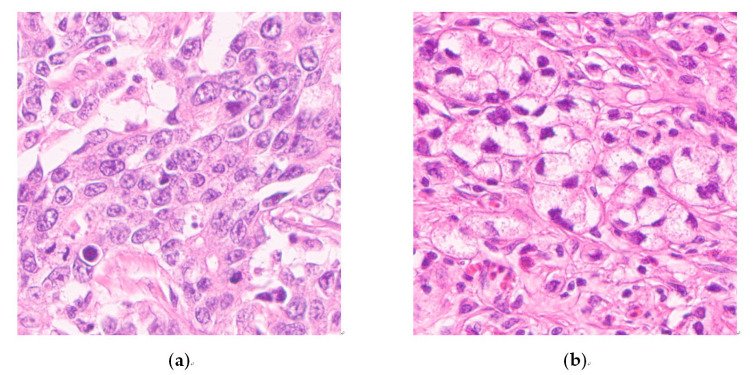
Representative images of cancer cell clusters of ≥5 carcinoma cells lacking glandular formation (magnification ×400). Image (**a**) is morphologically consistent with poorly differentiated carcinoma not otherwise specified, and image (**b**) is consistent with signet ring cell carcinoma.

When investigating PCC, we assessed PNCs, defined as cancer cell clusters of ≥5 carcinoma cells with a greater proportion of PCC-NOS than SRC, based on Kwon et al.’s study [23] as a presence/absence criterion to enhance reproducibility and interobserver consistency (K.Y. and S.H.L.) (Figure 2a,b). We applied the conventional PDC concept to intestinal-type EGC.

### 2.8. Statistical Analysis

The χ^2^ test, Fisher’s exact test, independent *t*-test and univariate logistic regression analyses were used to compare the absence and presence of LNM. Continuous data were converted to categorical variables using cutoff values, where the sum of sensitivity and specificity was maximized. Using multivariate logistic regression analysis, receiver operating characteristic (ROC) curves were calculated and the areas under the curves (AUCs) were obtained. Then, we used the Hanley–McNeil pairwise test to compare the effectiveness of PNC, TB and mTB by TB-YN, TB-ITBCC and total-TB in predicting LNM [31]. When performing multivariate logistic regression analysis, we used the variables that were significantly associated with LNM in univariate analysis. To avoid overfitting [32], we used indications of surgery after ER, which were made by combining the variables of tumor size, depth of invasion, ulcer and tumor types suggested in Japanese gastric cancer treatment guidelines [1], Age, sex, indication of surgery after ER and lymphatic and venous invasion were used as compounding factors (crude), then PNC, TB (YN, ITBCC, total) and mTB (YN, ITBCC, total) were added one by one to compounding factors and compared instead of using four factors as independent variables. Two-sided *p* values < 0.05 were considered statistically significant. All analyses were performed using SPSS software (version 20.0; IBM, Armonk, NY, USA).

## 3. Results

### 3.1. Clinicopathological Characteristics

A total of 289 patients with EGC were enrolled in the study. LNM was identified in 58 patients (20.1%). PNC, TB and mTB were significantly associated with LNM in overall EGC (Table 1), intramucosal EGC (Table S1) and undifferentiated-type dominant EGC (Table S2). Conversely, TB-equivalent SRC, (i.e., TB cells morphologically equivalent with SRC only) was not associated with LNM (Table 2).

There were no significant differences in sex, tumor location, gross type or ulcer between tumors with and without LNM. The average age between patients with and without LNM showed no significant difference, but according to analysis divided by the cutoff value where the sum of sensitivity and specificity was maximized (62 years), LNM was significantly higher in the older group. Tumor size, invasion depth and tumor differentiation were significantly associated with LN (Table 1). Among the various histological types, the prevalence of the mixed type was significantly higher in the LNM group than in the non-LNM group. As expected, lymphatic and venous invasions were significantly more common in the LNM group than in the non-LNM group (Table 1).

In our cohort, there was no cancer specific death. Death occurred in 38 out of 289 patients, as summarized in Table S3. In addition, recurrence in regional lymph node occurred in only one case (0.35%) after 13 months of ER.

### 3.2. Multivariate Regression Analysis of Risk Factors for LNM

**Figure 3:**
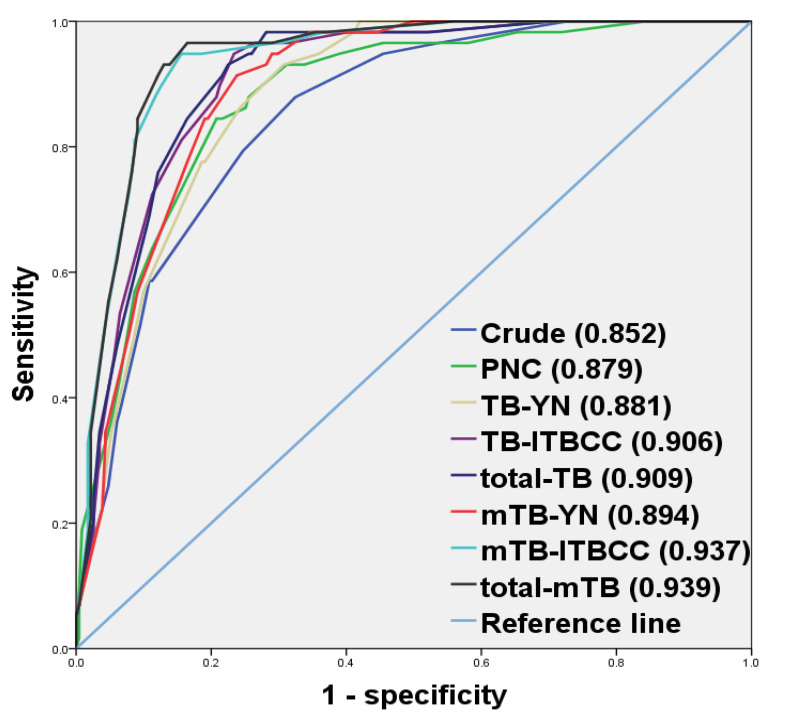
Comparison of receiver operating characteristic curves by multivariate logistic regression tests.

We found that mTB by total number was the most predictive factor for LNM. We found that mTB-ITBCC and total-mTB predicts LNM better than conventional TB-ITBCC and total-TB (*p* = 0.0049 and 0.0046, respectively). Between TB-YN and mTB-YN, there was no significant difference in predicting LNM (*p* = 0.0792). In addition, total-TB (*p* = 0.0226), total-mTB (*p* = 0.0011) and mTB-ITBCC (*p* = 0.0024) [24] were superior to TB-YN and mTB-YN for predicting LNM in EGCs but not in TB-ITBCC (*p* = 0.0560). Likewise, total-TB and total-mTB were not superior to TB-ITBCC (*p* = 0.7582) or mTB-ITBCC (*p* = 0.7212), respectively. Meanwhile, mTB-ITBCC (*p* = 0.0008) and total-mTB (*p* = 0.0004) had better LNM prediction capability than PNC (Table 3 and Figure 3).

All multivariate analysis models with TB and mTB revealed significant independent high-risk factors of LNM, including age (*p* = 0.004 to 0.047), lymphatic invasion (*p* < 0.001 to *p* = 0.018) and the use of TB or mTB (*p* < 0.001 to *p* = 0.002). In addition, the multivariate analysis model with PNC identified significant independent high-risk factors for LNM as indications of surgery after ER (*p* = 0.009), lymphatic invasion (*p* < 0.001), and PNC (*p* < 0.001) (Table S4).

## 4. Discussion

Our study demonstrated that mTB was more predictive of LNM than conventional TB (Table 3 and Figure 3). We also found that SRC-matched TB had no significant association with LNM; rather, it showed a greater tendency toward groups without LNM (Table 2). To the best of our knowledge, this is the first study to suggest the possibility that SRC-matched TB is not a risk factor for LNM and suggests the need to modify TB assessment in GC.

TB has been known to be associated with epithelial–mesenchymal transition (EMT) [12,33]. EMT is associated with invasiveness, metastasis and LNM [12]. The major molecular alteration in this process is decrease or absence of E-cadherin [34,35,36]. In addition, overexpression of TGF-β, deregulation of SMAD3/4 [37,38], loss of CD44, decrease of EpCAM [39,40] and activated SNAIL, TWIST, ZEB, RHOA [12,41] have been described. Shu et al. revealed genomic alterations of SRCC by whole genome sequencing. In their study, significantly mutated TP53, CDH1, PIK3CA, ERBB2 and LCE1F genes were noted, and no mutation of SMAD4, RHOA and ARID1A genes was reported [42]. Kwon et al. performed targeted genomic sequencing for GC with SRC-predominant and PCC-NOS-predominant type. They demonstrated that mutations in TP53, PIK3CA, BRAF, SMAD4 and RHOA were more concentrated in PCC-NOS-predominant than SRC-predominant type [23]. E-cadherin is known to be involved in the early onset of SRCC [22,43], but other molecules related to EMT, such as RHOA, SMAD4 and CD44 [23,41,42], are not. Therefore, as an EMT marker, assessing TB including SRC may not be appropriate for GC.

This study showed that total-TB and TB-ITBCC predicted LNM better than TB-YN methods. Total-TB seems to be superior to TB-ITBCC because total-TB reflects both PTB and ITB [12,24,25] and because it was difficult to specify an invasive front in the intrusive EGC. Actually, total-TB had a tendency to predict LNM better, but it was not statistically proven, and thus further research is required. Likewise, it might be superior to the TB-YN method because total-TB and TB-ITBCC could correct possible errors, such as tangentially sectioned tumor glands and histiocytes (Table 3 and Figure 3). The total-TB method would also be less laborious and more reproducible when the cutoff value (in this study, ≥5 tumor buds) is small.

In EGC, TB as a risk factor for LNM has been reported in a few studies and has been mostly applied to intestinal-type GCs [12,14] or studied regardless of pT stage [14,15,16]. We evaluated the usefulness of mTB in predicting LNM only in EGCs, regardless of intestinal/diffuse-type. Others have studied only submucosal EGC [17] and used only TB-YN [17,18]. For instance, Du et al. [17] and Gulluoglu et al. [18] reported the presence of TB as an independent LNM risk factor in 632 submucosal EGCs and 126 EGCs. We compared several TB assessment methods and all types of EGC, regardless of intramucosal/submucosal invasion. Olsen et al. [14] reported that high-grade TB (median number of tumor buds ≥1 in 10 ×200 fields) was associated with LNM and poor prognosis in 16 EGCs, Tanaka et al. [15] reported that high-grade TB (>10/×400 high-power fields) was associated with LNM in 65 EGCs, and Ulase et al. [16] reported that the presence of TB was a high risk factor for LNM in 57 EGCs.

Our study revealed that TB, regardless of the evaluation method, was the most predictive independent factor for LNM, for groups containing all kinds of EGCs, such as intramucosal, submucosal, intestinal-type and diffuse-type, in addition to other independent risk factors, such as lymphatic invasion and advanced age (Table 3 and Figure 3). TB was also proven to be a high-risk factor for LNM in intramucosal GC (Table S1) and “undifferentiated-type dominant” [1] EGC by the Japanese classification (Table S2). Moreover, by adding TB into conventional clinicopathological factors, the prediction model was much improved, far exceeding that of a previous result [44] (Table 3 and Figure 3). In addition, our study showed that SRC-equivalent TB was not associated with LNM (Table 2), and removing SRC from TB significantly increased the predictive power of LNM (Table 3 and Figure 3). SRCCs have shown better prognosis than intestinal-type EGC [12,14], and SRC is likely not related to EMT according to previous results [22,23,41,42,43]. We confirmed that total-TB and TB-ITBCC were superior to TB-YN for predicting LNM by comparing three TB assessment methods (Table 3 and Figure 3).

We also revealed PNC as a predictor for LNM; however, it had a lower predictive ability than TB (Table 3 and Figure 3). This may be because TB and PNC have different clinical impacts and/or mutations; however, it also could be because the SRC had not been completely removed from the PNC or because the grading method used only present/absent criteria. Therefore, further research is needed.

Lymphatic invasion is a well-known risk factor for LNM in EGC [14,15,16,17,18,45]. As demonstrated in this study, TB and lymphatic invasion were independent and the most important factors for predicting LNM (Table S4). When high-grade TB is observed in ER specimens, additional gastrectomy with lymph node dissection can be considered to increase recurrence and survival rates similar to the presence of lymphatic invasion [1].

Our study has several limitations, especially the relatively small number of LNM cases including intramucosal and undifferentiated-type dominant EGC compared to submucosal and intestinal-type EGC. Further research on a larger scale is required. However, since we collected and analyzed all EGCs in one institution over 10 years, our study cohort might well reflect the results of real-world practice. In addition, interobserver variations may exist in the interpretation of SRC. To overcome this weakness, in the present study, SRC was only recognized if it was morphologically consistent with the agreement of two pathologists (K.Y. and S.H.L.). When there was disagreement between the two pathologists, additional stains such as special staining for mucin were performed.

## 5. Conclusions

In conclusion, we demonstrated that excluding SRC from TB significantly increased its LNM prediction ability compared to conventional TB. In addition, we compared several TB assessment methods and revealed that total-TB and TB-ITBCC were superior to TB-YN for predicting LNM in EGCs. Counting all instances of TB after removing the SRC as proposed in our study may provide important information regarding treatment options for patients with EGCs.

## Figures and Tables

**Table 1:** Clinicopathological characteristics of all early gastric cancers.

Clinicopathologic Variable	Category	Absence of LNM *n* = 231 (79.9%)	Presence of LNM *n* = 58 (20.1%)	*p*
Lymph node number	*n* (mean ± SD)	38.7 ± 17.9	37.6 ± 14.1	0.646
Age	years old (mean ± SD)	62.5 ± 10.8	65.4 ± 10.4	0.066
	<62 *	105 (85.4%)	18 (14.6%)	0.047
	≥62 *	126 (75.9%)	40 (24.1%)	
Sex	Male	152 (79.2%)	40 (20.8%)	0.756
	Female	79 (81.4%)	18 (18.6%)	
Tumor location	Cardia	10 (83.3%)	2 (16.7%)	0.446
	Corpus/fundus	119 (82.6%)	25 (17.4%)	
	Antrum/angle/pylorus	102 (76.7%)	31 (23.3%)	
Gross type	I (protruding)	14 (77.8%)	4 (22.2%)	0.691
	IIa (flat elevated)	24 (80.0%)	6 (20.0%)	
	IIb (flat)	69 (82.1%)	15 (17.9%)	
	IIc (flat depressed)	106 (80.9%)	25 (19.1%)	
	III (excavated)	18 (69.2%)	8 (30.8%)	
Tumor size	mm (mean ± SD)	23.8 ± 16.6	35.6 ± 18.5	<0.001
Depth of invasion	pT1a	134 (95.7%)	6 (4.3%)	<0.001
	pT1b	97 (65.1%)	52 (34.9%)	
Tumor type	Differentiated ^†^	146 (83.0%)	30 (17.0%)	0.109
	Undifferentiated ^†^	85 (75.2%)	28 (24.8%)	
Histologic type (WHO)	Tubular	143 (84.6%)	26 (15.4%)	<0.001
	Papillary	4 (80.0%)	1 (20.0%)	
	Mucinous	0 (0%)	2 (100%)	
	SRCC	47 (94.0%)	3 (6.0%)	
	PCC-NOS	3 (60.0%)	2 (40.0%)	
	Mixed carcinoma	34 (58.6%)	24 (41.4%)	
Lauren classification	Intestinal type	148 (83.6%)	29 (16.4%)	<0.001
	Diffuse type	49 (90.7%)	5 (9.3%)	
	Mixed type	34 (58.6%)	24 (41.4%)	
Indications of surgery after ER ^‡^	Curability A	82 (97.6%)	2 (2.4%)	<0.001
	Curability B	25 (100.0%)	0 (0.0%)	
	Curability C	124 (68.9%)	56 (31.1%)	
Lymphatic invasion	Absent	207 (89.2%)	25 (10.8%)	<0.001
	Present	24 (42.1%)	33 (57.9%)	
Venous invasion	Absent	228 (81.4%)	52 (18.6%)	0.003
	Present	3 (33.3%)	6 (66.7%)	
PNC	Absent	135 (97.1%)	4 (2.9%)	<0.001
	Present	96 (64.0%)	54 (36.0%)	
TB-YN	Absent	123 (99.2%)	1 (0.8%)	<0.001
	Present	108 (65.5%)	57 (34.5%)	
TB-ITBCC	Low grade (Bd1)	168 (97.1%)	5 (2.9%)	<0.001
	High grade (Bd2, 3)	63 (54.3%)	53 (45.7%)	
total-TB	<5 *	165 (98.2%)	3 (1.8%)	<0.001
	≥5 *	66 (54.5%)	55 (45.5%)	
mTB-YN	Absent	146 (98.7%)	2 (1.3%)	<0.001
	Present	85 (60.3%)	56 (39.7%)	
mTB-ITBCC	Low grade (Bd1)	199 (97.1%)	6 (2.9%)	<0.001
	High grade (Bd2, 3)	32 (38.1%)	52 (61.9%)	
total-mTB	<5 *	199 (98.0%)	4 (2.0%)	<0.001
	≥5 *	32 (37.2%)	54 (62.8%)	

LNM, lymph node metastasis; SRCC, signet ring cell carcinoma; PCC-NOS, poorly cohesive carcinoma not otherwise specified; ER, endoscopic resection; PNC, poorly cohesive carcinoma not otherwise specified predominant cluster; TB-YN, presence or absence of tumor budding; TB-ITBCC, method proposed by International Tumor Budding Consensus Conference; total-TB, total numbers of tumor budding on a whole slide; mTB, modified tumor budding. * Cutoff values of age, total-TB and total-mTB were determined as where the sum of sensitivity and specificity were maximized. ^†^ According to the Japanese gastric cancer treatment guidelines 2018, the differentiated type includes papillary adenocarcinoma and well to moderately differentiated tubular adenocarcinoma, and the undifferentiated type includes poorly differentiated adenocarcinoma and PCC (including SRCC) [1]. ^‡^ This factor is made by combining the variables of tumor size, depth of invasion, ulcer, tumor type without resection margin involvement and lymphatic and venous invasion [1]. *p* < 0.05 was considered statistically significant. Continuous variables were compared using Student’s *t*-test, and nominal variables were compared by the χ^2^ test or Fisher’s exact test.

**Table 2:** Association of morphologically signet ring cell-equivalent tumor budding and lymph node metastasis in early gastric cancer with signet ring cell component and undifferentiated-type dominant. There are no associations with lymph node metastasis; therefore, excluding signet ring cell is necessary.

Clinicopathologic Variable	Category	In EGC with SRC Component		*p*
		Absence of LNM *n* = 82 (77.4%)	Presence of LNM *n* = 24 (22.6%)	
TB-YN, equivalent SRC	Absent	49 (71.0%)	20 (29.0%)	0.050
	Present	33 (89.2%)	4 (10.8%)	
TB-ITBCC, equivalent SRC	Low grade (Bd1)	53 (72.6%)	20 (27.4%)	0.131
	High grade (Bd2, 3)	29 (87.9%)	4 (12.1%)	
total-TB, equivalent SRC	<5 *	50 (71.4%)	20 (28.6%)	0.051
	≥5 *	32 (88.9%)	4 (11.1%)	
		**In Undifferentiated-Type Dominant EGC** ^†^		
		**Absence of LNM** ***n* = 85 (75.2%)**	**Presence of LNM** ***n* = 28 (24.8%)**	
TB-YN, equivalent SRC	Absent	53 (68.8%)	24 (31.2%)	0.034
	Present	32 (88.9%)	4 (11.1%)	
TB-ITBCC, equivalent SRC	Low grade (Bd1)	55 (69.6%)	24 (30.4%)	0.056
	High grade (Bd2, 3)	30 (88.2%)	4 (11.8%)	
total-TB, equivalent SRC	<5 *	54 (69.2%)	24 (30.8%)	0.034
	≥5 *	31 (88.6%)	4 (11.4%)	

EGC, early gastric carcinoma; SRC, signet ring cell; LNM, lymph node metastasis; TB-YN, presence or absence of tumor budding; TB-ITBCC, method proposed by International Tumor Budding Consensus Conference; total-TB, total numbers of tumor budding on a whole slide. * Cutoff values of age and total-TB were determined as where the sum of sensitivity and specificity was maximized. ^†^ According to the Japanese gastric cancer treatment guidelines 2018 [1]. *p* < 0.05 was considered statistically significant. All variables were compared by Fisher’s exact test.

**Table 3:** Association of morphologically signet ring cell-equivalent tumor budding and lymph node metastasis in early gastric cancer with signet ring cell component and undifferentiated-type dominant.

Clinicopathologic Variable	Univariate Analysis		Multivariate Analysis			
	Hazard Ratio (95% CI)	*p*	Hazard Ratio (95% CI)	*p*	AUC	Difference AUC
Age *	1.171 (1.003–3.421)	<0.001			0.852	
Sex	0.304 (0.466–1.608)	0.385				
Indications of surgery after ER ^†^	24.161 (5.759–101.362)	<0.001				
Lymphatic invasion	11.385 (5.827–22.245)	<0.001				
Venous invasion	8.769 (2.123–36.216)	0.003				
PNC	18.984 (6.651–54.185)	<0.001	7.597 (2.489–23.189)	<0.001	0.879	0.027
TB-YN	64.917 (8.839–476.757)	<0.001	24.358 (3.153–188.167)	<0.001	0.881	0.029
TB-ITBCC	28.267 (10.806–73.940)	<0.001	15.907 (5.407–46.804)	<0.001	0.906	0.054
total-TB	45.833 (13.852–151.653)	<0.001	25.495 (6.971–93.246)	<0.001	0.909	0.057
mTB-YN	48.094 (11.446–202.091)	<0.001	21.066 (4.753–93.374)	<0.001	0.894	0.042
mTB-ITBCC	53.896 (21.396–135.760)	<0.001	35.103 (12.109–101.756)	<0.001	0.937	0.085
total-mTB	83.953 (28.451–247.725)	<0.001	52.687 (15.687–179.973)	<0.001	0.939	0.087

AUC, area under receiver operating characteristic curve; ER, endoscopic resection; PNC, poorly cohesive carcinoma not otherwise specified predominant cluster; TB-YN, presence or absence of tumor budding; TB-ITBCC, method proposed by International Tumor Budding Consensus Conference; total-TB, total numbers of tumor budding on a whole slide; mTB, modified tumor budding; CI, confidence interval. * <62 years old vs. ≥62 years old. ^†^ This factor is made by combining the variables of tumor size, depth of invasion, ulcer, tumor type without resection margin involvement and lymphatic and venous invasion. Endoscopic curability A and B versus endoscopic curability C [1]. *p* < 0.05 was considered statistically significant. Logistic regression was performed and in multivariate analysis, age, sex, indications of endoscopic resection, lymphatic and venous invasion were used as compounding factors.

## Data Availability

Data can be made available upon reasonable request.

## Supplementary Materials

The following are available online at https://www.mdpi.com/article/10.3390/cancers13143405/s1, Table S1: Clinicopathological characteristics of intramucosal gastric cancers, Table S2: Clinicopathological characteristics of undifferentiated-type dominant early gastric cancers, Table S3: Summarized cause of death, Table S4: Multivariate logistic regression analysis of risk factors for lymph node metastasis.
