# Supplementary material for: Modified Tumor Budding as a Better Predictor of Lymph Node Metastasis in Early Gastric Cancer: Possible Real-World Applications
Source: Cancers (Basel). 2021 Jul 7;13(14):3405. doi: 10.3390/cancers13143405 (PMC8306932; doi:10.3390/cancers13143405)
Supplement: Supplementary file 1 [file cancers-13-03405-s001.zip › cancers-1272746-supplementary.pdf]

Table S1. Clinicopathological characteristics of intramucosal gastric cancers.

Clinicopathologic Variable	Category	Absence of LNM<br>*n* = 134 (95.7%)	Presence of LNM<br>*n* = 6 (4.3%)	*p*
Lymph node number	*n* (mean ± SD)	38.1±15.7	46.7±20.8	0.197
Age	years old (mean ± SD)	62.5±11.5	65.7±9.6	0.508
	<62*	59 (98.3%)	1 (1.7%)	0.186
	≥62*	75 (93.8%)	5 (6.2%)	
Sex	Male	85 (93.4%)	6 (6.6%)	0.091
	Female	49 (100.0%)	0 (0.0%)	
Tumor location	Cardia	4 (100.0%)	0 (0.0%)	
	Corpus/fundus	72 (98.6%)	1 (1.4%)	0.154
	Antrum/angle/pylorus	58 (92.1%)	5 (7.9%)	
Gross type	I (protruding)	5 (100.0%)	0 (0.0%)	0.454
	IIa (flat elevated)	13 (100.0%)	0 (0.0%)	
	IIb (flat)	47 (94.0%)	3 (6.0%)	
	IIc (flat depressed)	64 (97.0%)	2 (3.0%)	
	III (excavated)	5 (83.3%)	1 (16.7%)	
Tumor size	mm (mean ± SD)	20.5±15.4	40.2±26.8	0.004
Ulcer	Absent	101 (98.1%)	2 (1.9%)	0.042
	Present	33 (89.2%)	4 (10.8%)	
Tumor type†	Differentiated	85 (96.6%)	3 (3.4%)	0.670
	Undifferentiated	49 (94.2%)	3 (5.8%)	
Histologic type (WHO)	Tubular	81 (96.4%)	3 (3.6%)	0.162
	Papillary	1 (100.0%)	0 (0%)	
	SRCC	33 (97.1%)	1 (2.9%)	
	PCC-NOS	1 (50.0%)	1 (50.0%)	
	Mixed carcinoma	18 (94.7%)	1 (5.3%)	
Lauren classification	Intestinal type	82 (96.5%)	3 (3.5%)	0.716
	Diffuse type	34 (94.4%)	2 (5.6%)	
	Mixed type	18 (94.7%)	1 (5.3%)	
Indications of surgery after ER‡	Curability A	82 (97.6%)	2 (2.4%)	0.076
	Curability B	18 (100.0%)	0 (0.0%)	
	Curability C	34 (89.5%)	4 (10.5%)	
Lymphatic invasion	Absent	133 (95.7%)	6 (4.3%)	0.832
	Present	1 (100.0%)	0 (0.0%)	
Venous invasion	Absent	134 (95.7%)	6 (4.3%)	NA
	Present	0 (0.0%)	0 (0.0%)	
PNC	Absent	102 (99.0%)	1 (1.0%)	0.005
	Present	32 (86.5%)	5 (13.5%)	
TB-YN	Absent	90 (100.0%)	0 (0.0%)	0.002
	Present	44 (88.0%)	6 (12.0%)	
TB-ITBCC	Low grade (Bd1)	109 (98.2%)	2 (1.8%)	0.017
	High grade (Bd2, 3)	25 (86.2%)	4 (13.8%)	
total-TB	<5*	108 (99.1%)	1 (0.9%)	0.002
	≥5*	26 (83.9%)	5 (16.1%)	
mTB-YN	Absent	107 (100.0%)	0 (0.0%)	<0.001
	Present	27 (81.8%)	6 (18.2%)	
mTB-ITBCC	Low grade (Bd1)	127 (98.4%)	2 (1.6%)	<0.001
	High grade (Bd2, 3)	7 (63.6%)	4 (36.4%)	
total-mTB	<5*	129 (99.2%)	1 (0.8%)	<0.001

	≥5*	5 (50.0%)	5 (50.0%)

LNM, lymph node metastasis; SRCC, signet ring cell carcinoma; PCC-NOS, poorly cohesive carcinoma-not otherwise specified; NA, not available; ER, endoscopic resection; PNC, poorly cohesive carcinoma-not otherwise specified predominant cluster; TB-YN, presence and absence of tumor budding; TB-ITBCC, method proposed by International Tumor Budding Consensus Conference; total-TB, total numbers of tumor budding on a whole slide; mTB, modified tumor budding. *Cut off values of age, total-TB and total-mTB were determined at where the sum of sensitivity and specificity were maximized. †According to the Japanese gastric cancer treatment guidelines 2018. ‡This factor is made by combining the variables of tumor size, depth of invasion, ulcer, and tumor type without resection margin involvement, lymphatic and venous invasion. $p < 0.05$, was considered statistically significant. Continuous variables were compared using Student's t-test and nominal variables were compared by Fisher's exact test.

Table S2. Clinicopathological characteristics of undifferentiated-type dominant early gastric cancers.

Clinicopathologic Variable	Category	Absence of LNM n = 85 (75.2%)	Presence of LNM n = 28 (24.8%)	p
Lymph node number	n (mean ± SD)	39.7±17.3	38.1±13.6	0.646
Age	years old (mean ± SD)	58.5±10.8	64.5±10.8	0.013
	<62*	51 (85.0%)	9 (15.0%)	0.016
	≥62*	34 (64.2%)	19 (35.8%)	
Sex	Male	42 (71.2%)	17 (28.8%)	0.299
	Female	43 (79.6%)	11 (20.4%)	
Tumor location	Cardia	5 (83.3%)	1 (16.7%)	0.265
	Corpus/fundus	52 (80.0%)	13 (20.0%)	
	Antrum/angle/pylorus	28 (66.7%)	14 (33.3%)	
Gross type	I (protruding)	2 (40.0%)	3 (60.0%)	0.320
	IIa (flat elevated)	3 (75.0%)	1 (25.0%)	
	IIb (flat)	34 (71.0%)	8 (19.0%)	
	IIc (flat depressed)	43 (75.4%)	14 (24.6%)	
	III (excavated)	3 (60.0%)	2 (40.0%)	
Tumor size	mm (mean ± SD)	27.5±19.9	37.5±19.7	0.394
Depth of invasion	pT1a	49 (94.2%)	3 (5.8%)	<0.001
	pT1b	36 (59.0%)	25 (41.0%)	
Histologic type (WHO)	Tubular	7 (77.8%)	2 (22.2%)	<0.001
	Mucinous	0 (0.0%)	2 (100.0%)	
	SRCC	47 (94.0%)	3 (6.0%)	
	PCC-NOS	4 (66.7%)	2 (33.3%)	
	Mixed carcinoma	27 (58.7%)	19 (41.3%)	
Lauren classification	Intestinal type	9 (90.9%)	4 (30.8%)	0.001
	Diffuse type	49 (90.7%)	5 (9.3%)	
	Mixed type	27 (58.7%)	19 (41.3%)	
Indications of surgery after ER†	Curability B	18 (100.0%)	0 (0.0%)	0.006
	Curability C	67 (70.5%)	28 (29.5%)	
Lymphatic invasion	Absent	75 (87.2%)	11 (12.8%)	<0.001
	Present	10 (37.0%)	17 (63.0%)	
Venous invasion	Absent	84 (77.1%)	25 (22.9%)	0.046
	Present	1 (25.0%)	3 (75.0%)	
PNC	Absent	53 (96.4%)	2 (3.6%)	<0.001
	Present	32 (55.2%)	26 (44.8%)	
TB-YN	Absent	30 (96.8%)	1 (3.2%)	0.001
	Present	55 (67.1%)	27 (32.9%)	
TB-ITBCC	Low grade (Bd1)	37 (94.9%)	2 (5.1%)	<0.001
	High grade (Bd2, 3)	48 (64.9%)	26 (35.1%)	
total-TB	<5*	37 (97.4%)	1 (2.6%)	0.001
	≥5*	48 (64.0%)	27 (36.0%)	
mTB-YN	Absent	52 (96.3%)	2 (3.7%)	<0.001
	Present	33 (55.9%)	26 (44.1%)	
mTB-ITBCC	Low grade (Bd1)	65 (95.6%)	3 (4.4%)	<0.001
	High grade (Bd2, 3)	20 (44.4%)	25 (55.6%)	
total-mTB	<5*	65 (97.0%)	2 (3.0%)	<0.001
	≥5*	20 (43.5%)	26 (56.5%)	

LNM, lymph node metastasis; SRCC, signet ring cell carcinoma; PCC-NOS, poorly cohesive carcinoma-not otherwise specified; ER, endoscopic resection; PNC, poorly cohesive carcinoma-not otherwise specified predominant cluster; TB-YN, presence and absence of tumor budding; TB-ITBCC, method proposed by International Tumor Budding Consensus Conference; total-TB, total numbers of tumor budding on a whole slide; mTB, modified tumor budding. *Cut off values of age, total-TB and total-mTB were determined at where the sum of sensitivity and specificity were maximized. †This factor is made by combining the variables of tumor size,

depth of invasion, ulcer, and tumor type without resection margin involvement, lymphatic and venous invasion. $p < 0.05$, was considered statistically significant. Continuous variables were compared using Student's t-test and nominal variables were compared by χ^2 test or Fisher's exact test.

Table S3. Summarized cause of death.

Category	Cause of death	*n*	Total
Cancer specific death		0	0
Other malignancy	Prostate cancer	2	10
	Lung cancer	2	
	Colon cancer	1	
	Cholangiocarcinoma	1	
	Brain tumor	1	
	Pancreatic cancer	1	
	Vocal cord cancer	1	
	Urothelial cancer	1	
Medical conditions	Sepsis	7	27
	Pneumonia	6	
	Myocardial infarction	4	
	Cerebrovascular accident	3	
	Anastomosis leakage	2	
	Dementia	2	
	Deep vein thrombosis	1	
	Liver cirrhosis	1	
	Alcoholic ketoacidosis	1	
Traffic accident		1	1

Table S4. Multivariate logistic regression analysis of risk factors for lymph node metastasis.

Clinicopathologic Variable	Crude		PNC		TB-YN		TB-ITBCC		total-TB		mTB-YN		mTB-ITBCC		total-mTB	
	**Hazard ratio (95% CI)**	***p***	**Hazard ratio (95% CI)**	***p***	**Hazard ratio (95% CI)**	***p***	**Hazard ratio (95% CI)**	***p***	**Hazard ratio (95% CI)**	***p***	**Hazard ratio (95% CI)**	***p***	**Hazard ratio (95% CI)**	***p***	**Hazard ratio (95% CI)**	***p***
Age*	2.094 (1.027-4.269)	0.042	1.871 (0.882-3.971)	0.103	2.130 (1.010-4.496)	0.047	2.615 (1.173-5.832)	0.019	2.722 (1.216-6.097)	0.015	2.391 (1.102-5.186)	0.024	3.995 (1.573-10.144)	0.004	3.443 (1.371-8.643)	0.008
Sex	0.783 (0.377-1.624)	0.511	1.017 (0.467-2.217)	0.965	5.392 (1.139-25.515)	0.034	4.066 (0.819-20.190)	0.086	0.571 (0.254-1.282)	0.174	5.193 (1.076-25.069)	0.040	0.834 (0.332-2.091)	0.698	0.962 (0.378-2.450)	0.935
Indications of surgery after ER†	12.607 (2.878-55.219)	0.001	7.509 (1.662-33.937)	0.009	5.352 (1.156-24.782)	0.032	3.393 (0.690-16.697)	0.133	3.071 (0.614-15.353)	0.172	5.915 (1.265-27.669)	0.024	2.988 (0.573-15.583)	0.194	2.100 (0.371-11.877)	0.402
Lymphatic invasion	5.746 (2.807-11.762)	<0.001	4.130 (1.947-8.759)	<0.001	4.360 (2.062-9.220)	<0.001	4.685 (2.085-10.526)	<0.001	4.191 (1.852-9.481)	0.001	3.715 (1.717-8.037)	0.001	3.686 (1.473-9.225)	0.005	3.093 (1.217-7.858)	0.018
Venous invasion	2.835 (0.580-13.866)	0.198	2.371 (0.447-12.576)	0.310	2.166 (0.454-10.328)	0.332	2.625 (0.363-19.000)	0.339	2.929 (0.383-22.396)	0.301	1.713 (0.362-8.099)	0.497	1.438 (0.174-11.857)	0.736	1.630 (0.194-13.683)	0.653
PNC			7.597 (2.489-23.189)	<0.001												
TB-YN					24.358 (3.153-188.167)	0.002										
TB-ITBCC							15.907 (5.407-46.804)	<0.001								
total-TB									25.495 (6.9714-93.246)	<0.001						
mTB-YN											21.066 (4.753-93.374)	<0.001				
mTB-ITBCC													35.103 (12.109-101.756)	<0.001		
total-mTB															52.687 (15.424-179.973)	<0.001

ER, endoscopic resecion; PNC, poorly cohesive carcinoma-not otherwise specified predominant cluster; TB-YN, presence and absence of tumor budding; TB-ITBCC, method proposed by International Tumor Budding Consensus Conference; total-TB, total numbers of tumor budding on a whole slide; mTB, modified tumor budding. *<62 years old versus ≥62 years old. †This factor is made by combining the variables of tumor size, depth of invasion, ulcer, and tumor type without resection margin involvement, lymphatic and venous invasion. Endoscopic curability A and B versus endoscopic curability C. ‡According to the Japanese gastric cancer treatment guidelines 2018, differentiated type vs. undifferentiated type. $p < 0.05$, was considered statistically significant. When performing multivariate logstic regression test, age, sex, indications of endoscopic resecion, lymphatic and venous invasion were used for compounding factors.
